# Epigenetic Control of the Genome—Lessons from Genomic Imprinting

**DOI:** 10.3390/genes5030635

**Published:** 2014-08-14

**Authors:** Bjorn T. Adalsteinsson, Anne C. Ferguson-Smith

**Affiliations:** Department of Genetics, University of Cambridge, Cambridge CB2 3EH, UK; E-Mail: bta26@cam.ac.uk

**Keywords:** Epigenetics, imprinting, gene expression, gene regulation, CTCF, long non-coding RNA, histone modifications, DNA methylation

## Abstract

Epigenetic mechanisms modulate genome function by writing, reading and erasing chromatin structural features. These have an impact on gene expression, contributing to the establishment, maintenance and dynamic changes in cellular properties in normal and abnormal situations. Great effort has recently been undertaken to catalogue the genome-wide patterns of epigenetic marks—creating reference epigenomes—which will deepen our understanding of their contributions to genome regulation and function with the promise of revealing further insights into disease etiology. The foundation for these global studies is the smaller scale experimentally-derived observations and questions that have arisen through the study of epigenetic mechanisms in model systems. One such system is genomic imprinting, a process causing the mono-allelic expression of genes in a parental-origin specific manner controlled by a hierarchy of epigenetic events that have taught us much about the dynamic interplay between key regulators of epigenetic control. Here, we summarize some of the most noteworthy lessons that studies on imprinting have revealed about epigenetic control on a wider scale. Specifically, we will consider what these studies have revealed about: the variety of relationships between DNA methylation and transcriptional control; the regulation of important protein-DNA interactions by DNA methylation; the interplay between DNA methylation and histone modifications; and the regulation and functions of long non-coding RNAs.

## 1. A Primer on Epigenetics, DNA Methylation and Histone Modifications

Epigenetic modifications perform three main functions in mammalian cells: they contribute to the control of chromosome architecture ensuring stability and appropriate segregation of chromosomes during mitosis; they contribute to regulation of the silencing and inaccessibility of repetitive elements and endogenous retroelements; and they can initiate and maintain the activity and repression of individual genes or clusters of genes. Here we focus on the role of epigenetic modifications in the control of mammalian transcription and the contribution of genomic imprinting studies to our understanding of epigenetic mechanisms.

In mammals, the different cells that make up an organism generally contain the same DNA yet their cellular morphology and function can vary greatly. This is largely a result of differential gene expression, which is developmentally regulated and can then be maintained after repeated cell divisions. The maintenance of expression states/levels requires heritable information to be passed through cell division to ensure propagation in each daughter cell, and it is this information that has been termed epigenetic. Further, cells are subject to dynamic changes in gene expression, dependent, for example, on intrinsic and extrinsic cues, which can be mediated through epigenetic processes. Epigenetic mechanisms include DNA methylation and post translational modifications to core histones. Other related components have been proposed as epigenetic such as non-coding RNAs (ncRNAs) and nucleosomal positioning, however these might also be considered mediators and/or facilitators of epigenetic states. The characterization and mapping of genome-wide epigenetic modifications represent an ever increasing field of research. These studies are revealing genome-wide patterns of epigenetic regulation that not only have confirmed many of the conclusions suggested from more traditional experimental approaches in model systems but also allow for the generation of new hypotheses that await experimental testing. One model system that contributed a foundation for these studies is the process of genomic imprinting.

DNA methylation is a process whereby a methyl (CH_3_) group is added most commonly to a cytosine in DNA. In mammals it is generally found at CpG dinucleotides and can be correlated with gene repression in a variety of ways (discussed in more detail below). CpG sites are generally depleted in the genome, apart from stretches of DNA called CpG islands where CpG density is high. CpG islands can be concentrated at gene promoters and are generally unmethylated. CpG sites outside CpG islands are generally methylated (reviewed in [1,2])—resulting in a genome-wide methylation pattern that can be described as roughly bimodal. Acquisition of DNA methylation is catalyzed by a family of DNA methyltransferases (DNMTs, reviewed in [3]). DNMT1 has affinity for hemi-methylated DNA and is responsible for maintaining methylation after DNA replication and DNMT3A and DNMT3B catalyze *de novo* DNA methylation while the DNA methyltransferase homologue, DNMT3L acts as a cofactor and has no methyltransferase activity.

Waves of DNA methylation loss and acquisition are orchestrated during embryonic development. After fertilization the two parental genomes are mostly stripped of their epigenetic marks, a process that presumably “resets” the genome to a naive state applicable for pluripotency (DNA methylation at certain sequences in imprinted loci are among few genomic regions to “escape” this demethylation, see details in Section 2). Around blastocyst implantation *de novo* methylation then occurs and, to our knowledge, no further genome wide erasure/acquisition waves occur in somatic cells. Another wave of genome-wide reprogramming occurs in primordial germ cells (this time DNA methylation at imprinted loci is also lost, see details in Section 2); erasure of DNA methylation commences in the embryonic germline after embryonic day 7.5 (E7.5) in the mouse and progressive *de novo* methylation follows at E12.5 in prospermatogonia of male embryos, but occurs after birth in oocytes of female embryos (reviewed in [4]). This germline epigenetic reprogramming is required for generating functional germ cells and failure to do this appropriately usually results in infertility or developmentally abnormal embryos that die during gestation [5,6].

Covalent post-translational modifications to core histones (histone modifications henceforth) can impact the conformation of the nucleosome-nucleosome architecture within chromatin and influence its function such that some modifications are associated with an active chromatin state and others with a repressive state (for extensive review refer to [7]). The full repertoire of histone modifications is unknown, but is complex, with some specific amino acid residues influencing the ability of others to be modified, and some sites having the potential to be modified in multiple different ways. It is currently unclear whether many of the modifications truly are epigenetically heritable in a replication-dependent manner, like DNA methylation. Lysine methylation and lysine acetylation are among the best characterized histone modifications whose correlations with gene activity and repression have been extensively studied. Furthermore, enzymes involved in “writing” and “erasing” these epigenetic marks have been identified and characterized; histone lysine methyltransferases deposit methyl groups to lysine, and histone lysine demethylases remove them. Histone acetyltransferases (KATs) and histone deacetylases (HDACs) deposit and remove acetyl groups, respectively. Generally, regions with acetylated histones are associated with gene activity and regions devoid of acetylated histones are repressed, while associations between histone methylation and gene transcription are more site specific; histone 3 lysine 4 (H3K4) and H3K36 methylation are for example found on expressed genes while H3K9 methylation is associated with repressed genes. Their distribution in the genome can be associated with certain genomic motifs, e.g., gene regions such as promoters or open reading frames (ORFs), or intergenic regions such as repeats. For example, H3K4me3 (me3 denotes tri-methylated) is found at the promoters of active genes, whilst H3K4me1 is associated with enhancers, H3K20me3 is found at repressed repeat regions, and H3K9me3 at promoters of repressed genes, retroelements, imprinted loci and at pericentromeric repeat regions.

## 2. Genomic Imprinting and Targeting DNA Methylation

Genomic imprinting is a process causing the mono-allelic expression of a specific subset of mammalian genes in a parental origin specific manner (reviewed in [8,9])—*i.e.*, genes that are expressed either from the paternally inherited chromosome or from the maternally inherited chromosome (paternal allele and maternal allele henceforth) are imprinted. The non-equivalence of parental genomes in mammals was discovered in 1984 [10,11], and individual imprinted genes were first discovered in 1991 (reviewed in [8]). Today, over 100 imprinted genes have been identified, most of which are organized in clusters and are regulated in a coordinated manner by a single imprinting control region (ICR) [9]. Most clusters contain at least one non coding gene and multiple protein coding genes, whose functions regulate embryonic development, placentation and a range of post-natal processes.

Epigenetic mechanisms allow the transcriptional machinery of the cell to distinguish the two parental chromosomes at imprinted loci and hence provide an important paradigm for understanding epigenetic control of gene activity and repression. Specifically, the discovery of differences in DNA methylation in the same place on the two parental chromosomes suggested the importance of epigenetic mechanisms in regulating imprinting [12,13] and the potential for epigenetic control in a wider context. The identification of imprinting control regions and their validation genetically as functional elements essential for the imprinting of multiple genes in *cis*, elucidated imprinting control. The loss of imprinting after targeted deletion of DNMT1 proved that DNA methylation was required for imprinting [14]. Importantly, in the absence of DNMT1, some imprinted genes were activated but others became repressed, an indication that methylation could impact activity as well as repression.

The acquisition of methylation at ICRs occurs in the germ line *de novo* by DNMT3A and DNMT3L with a small number of ICRs becoming methylated in sperm cells, and the majority acquiring methylation in oocytes—paternal and maternal ICRs, respectively. It is of interest that paternal ICRs are always located in intergenic regions while maternal ICRs are located at promoter sequences. Importantly, erasure of imprints occurs in the wave of demethylation that occurs in the primordial germ cells. However, in order to retain the memory of the parental origin that is subsequently established after that reprogramming, imprints must be retained during the post-fertilization epigenetic reprogramming phase [4]. Interestingly, other regions of the genome seem refractory to zygotic reprogramming [15] though these are not necessarily parent-specific or retained like imprints during development. The relationship, if any, of these regions to ICRs remains unclear. In addition to the ICR, other differentially methylated regions (DMRs) are located at some imprinted clusters, but a notable difference between ICRs and these DMRs is that differential methylation of the latter is not germline established, but rather is acquired post-fertilization. In all cases, these so-called secondary DMRs—to distinguish them from regions such as ICRs that acquire differential methylation in the germline—require the ICR for their establishment. The mechanisms through which ICRs control gene expression in their respective clusters are diverse and remain the subject of active research, including analysis of regulation by ncRNAs and of the relationships between DNA methylation and histone and non-histone proteins.

**Figure 1:**
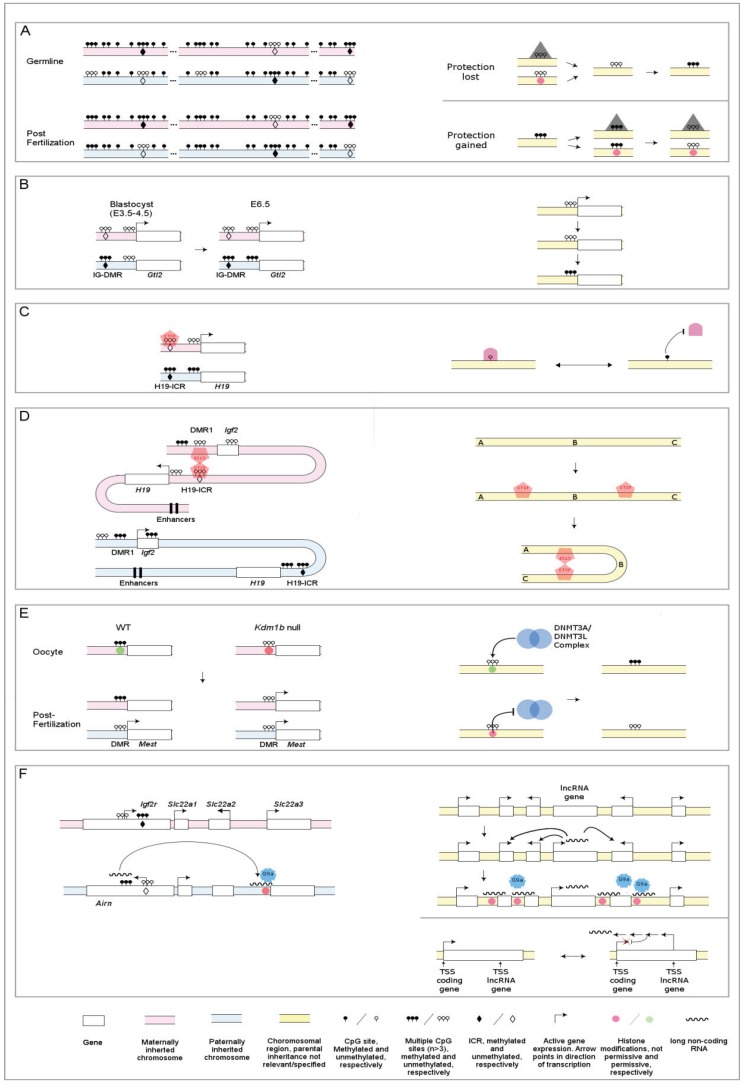
Regulatory epigenetic phenomena at imprinted loci. On the left are examples of various epigenetic mechanisms as observed in imprinted loci, and on the right models are presented of how those principles may apply more generally.

Both in imprinted and non-imprinted contexts, little is known about why certain DNA sequences become methylated and not others, or how this may change dynamically within a sequence such as a particular CpG island at a gene promoter. Most likely, it is a process that must be targeted in some manner. Targeting of the DNA methylation machinery has received much attention and efforts made to identify intrinsic sequence specificities of DNMTs and their cofactors. It has thus generally been assumed that the acquisition of methylation represents the “active” process in establishing differential methylation. However, recent studies on DMRs in the germlines and their propagation after fertilization suggest it might also be protection from DNA methylation and maintenance at methylated regions that determine differential methylation (Figure 1A, reviewed in [16]): Rather than appearing as discrete methylated sequences in otherwise unmethylated regions, maternal ICRs (which represent the vast majority of ICRs) are surrounded by methylation at both flanks. In contrast, these ICRs are unmethylated in sperm but are also flanked by methylation at surrounding sequences, suggesting that DNA methylation may be the “default” state and that it is protection from methylation at the ICRs, and perhaps other non-imprinted sequences as well, that establishes their differential methylation. Furthermore, in the germline, far more sequences are differentially methylated between oocytes and sperm than the ICRs; recent genome-wide studies suggest they are in the counts of thousands in oocytes and hundreds in sperm [15,17,18]. In contrast to ICRs these sequences generally lose methylation after fertilization, suggesting targeted maintenance of DNA methylation at specific sequences is essential for the germline-derived differential methylation of imprinted loci. Hence perhaps, loss of maintenance, in addition to active removal of DNA methylation at non-imprinted loci, contributes to the mechanism through which demethylation occurs in somatic cells. KRAB zinc finger proteins (ZFP) represent a family of over 350 tetrapod-specific genes whose functions remain poorly understood. They bind DNA and have previously been shown to recruit the repressive chromatin machinery in a site-specific manner. One of these KRAB-ZFPs, ZFP57, has been shown to be required to maintain the DNA methylation memory at imprints during post-fertilization reprogramming when the bulk of the genome is changing its epigenetic state [19]. ZFP57 binds methylated DNA and is thought to recruit methyltransferases to imprinting control regions hence preventing them from loss of their imprints.

## 3. DNA Methylation and Gene Repression—The Chicken or the Egg?

### 3.1. DNA Methylation Correlates with Repression

The correlation between DNA methylation and gene repression was noted in several experiments assaying viral and endogenous gene expression in mammalian, frog and sea urchin cells in the late 1970s and early 80s [20,21,22,23,24,25,26,27,28,29,30]. Experiments were conducted to determine whether the observed relationship was purely correlational, or whether DNA methylation functionally regulated gene expression. This was, however, challenging, but the strong evidence in many different contexts, showing that hypomethylated regions were associated with activity and hypermethylated regions refractory to transcription, suggested that absence of DNA methylation may be necessary though not sufficient for transcription. Vardimon *et al*. injected bacterial plasmids containing *in vitro* methylated or unmethylated DNA encoding a viral gene into frog oocyte nuclei [31]. They observed maintenance of the respective methylation states over a 24 h period, and expression of the gene in oocytes that were injected with unmethylated DNA but not in those that were injected with methylated DNA [31]. In a similar experiment, Stein *et al*. transfected *in vitro* methylated or unmethylated plasmids containing the *Aprt* (adenine phosphoribosiltransferase) gene into cultured *Aprt* null mouse cells. They observed maintenance of the respective *Aprt* methylation states after integration into the endogenous genome over several cell divisions for both unmethylated and methylated plasmids, and that integration of the unmethylated but not the methylated gene rescued the *Aprt* null phenotype, suggesting methylation of the gene was associated with inhibition of its transcription [32].

Correlations between gene expression and DNA methylation have been assessed at CpG sites across whole chromosomes or the whole genome. Consistent with the earlier studies, DNA methylation of promoter sequences, though rare at CpG island promoters, was observed to correlate with gene repression [33,34,35]. The functional role of DNA methylation in repressing gene expression is further suggested by results from studies in which the genes encoding the DNA methyltransferases are deleted conditionally in various cell lineages. Generally, the loss of DNMTs results in dysregulation of multiple genes, with a trend towards gene activation rather than silencing, again suggesting that DNA methylation represses gene expression (reviewed in [36]). Furthermore, treatment of cells *in vivo* with the DNA methyltransferase inhibitor 5-Azacytidine was shown to result in gene activation in several experiments in the 1980s, with concomitant loss of DNA methylation (reviewed in [37]). Together all these findings have led to the general assumption that loss and acquisition of DNA methylation at a gene promoter results in gene activation and silencing, respectively, but none actually proved that the acquisition of DNA methylation itself causes the gene silencing in all contexts.

### 3.2. DNA Methylation as a Consequence of Transcriptional Silencing

Studies of the temporal onset of mono-allelic expression of imprinted genes and the acquisition of differential methylation at secondary DMRs during mouse development indicate that DNA methylation can be acquired *after* gene repression (Figure 1B). The imprinted genes *Gtl2*, *Cdkn1C*, *H19* and *Igf2r* each contain a secondary DMR in their promoters, which become differentially methylated days *after* their mono-allelic expression is observed (summarized in [38]). Generally, mono-allelic expression of these genes is initiated around the morula or blastocyst stage (E3.5-4.5), while differential methylation of the respective secondary DMR occurs after E6.5 [13,38,39,40,41,42]. In the most extreme case, *Igf2r* is mono-allelically expressed from the maternal allele from E6.5 onward but the silent paternal allele only becomes methylated at or after E15.5 [13,42]. It is reasonable to assume that this temporal relationship, where methylation is acquired as a consequence of gene repression, also applies to non-imprinted genes (Figure 1B)*.* In particular, is has recently been shown that DNA methylation levels are secondary to the binding of transcription factors; Stadler *et al.* [43] identified multiple clusters of CpG sites that have low to intermediate levels of methylation, 10%–50%, in mouse embryonic stem (ES) cells. These low methylated regions (LMRs) are likely distal regulatory regions, and are bound by various transcription factors. Scrambling binding sites for the insulator protein CTCF or knocking out the transcription factor REST led to increased methylation at the LMRs. Furthermore, reintroduction of REST into the *REST*^−/−^ cells reverted the methylation status of the LMRs to the normal low levels [43]. These findings suggest DNA methylation may not have a direct role in silencing gene expression in all situations. In such cases DNA methylation might rather be acquired after gene silencing to maintain the repressed state or as a secondary readout of other mechanisms of genome control. Nonetheless, there are situations where acquisition of DNA methylation unquestionably does regulate gene expression, notably at the germline DMRs of imprinted genes [1,8,9,14,15,16].

## 4. How Does DNA Methylation Confer Effects on Gene Expression?

### 4.1. Proteins Attracted and Repelled

In situations where DNA methylation does indeed direct gene repression there are currently two model mechanisms that are generally acknowledged [1,44]: First, DNA methylation can attract proteins that bring about gene repression through recruitment of chromatin modifiers. A group of proteins, collectively referred to as methyl binding proteins (MBPs) have been characterized and shown to specifically bind to methylated, but not unmethylated, DNA [44,45,46,47,48,49]. MBPs are known to interact with histone modifiers such as HDACs, e.g., in forming complexes, such as the nucleosome remodeling deacetylase (NuRD) complex, which through their histone deacetylase activity and subsequent chromatin condensation bring about gene repression [50,51,52,53,54,55]. Secondly, certain proteins may interact with DNA in a methylation dependent manner. Here, DNA methylation may be refractory to the binding of proteins, such as transcription factors or other regulatory proteins [56,57,58], that are necessary for gene expression (Figure 1C). For this latter model, the best characterized example is the regulation of CTCF binding at the imprinted H19/Igf2 cluster *via* differential DNA methylation on the two parental alleles (reviewed in [1]).

### 4.2. Regulation of CTCF Binding at the H19/Igf2 Imprinted Cluster; the Insulator Mechanism

In the H19/Igf2 imprinted cluster, the protein coding gene *Igf2* is expressed from the paternally inherited allele [59]. This expression pattern is dependent on the regional ICR [60], on its differential methylation [12,14,61] and on the insulator protein CTCF binding to the ICR. On the unmethylated maternal allele, CTCF can bind, while its binding is inhibited on the methylated paternally inherited chromosome [62,63,64,65]—thus CTCF binding to DNA is methylation-sensitive (Figure 1C). *Igf2* and a downstream non-coding RNA gene, *H19,* share enhancers that are located at the 3' end of *H19* [66,67] and the parental specific expression of *Igf2* and *H19* are ultimately determined by interaction with these sequences; on the paternally inherited chromosome, *Igf2*-enchancer interaction is possible and the gene is expressed. On the maternally inherited chromosome this contact is blocked by CTCF binding to the ICR and this facilitates enhancer interaction with a now active *H19* instead, and also results in *Igf2* repression.

What is the mechanism of CTCF’s enhancer blocking activity? The current model (reviewed in [68]) suggests that in the H19/Igf2 cluster, chromatin loop formation on the maternal allele spatially inhibits enhancer interaction with *Igf2.* The process appears to depend on three elements; dimerization, CTCF binding to more than one region and physical contact between these neighboring sites via CTCF interaction [69,70,71,72,73,74]. The model suggests that on the unmethylated maternally inherited chromosome, CTCF binds to the ICR and also to an upstream somatic DMR located 5' of *Igf2*. Binding does not occur at the paternal allele where methylation inhibits the binding. On the maternal allele ICR-DMR contact is made possible by CTCF dimerization bringing together the two distinct loci, and because they flank *Igf2*, the gene is ‘looped out’ (Figure 1D). Further chromatin contacts within the cluster, some facilitated by CTCF, then result in physical separation between the *Igf2* loop and the enhancers. Recently cohesins have been shown to bind to over half of CTCF binding sites in the genome, including in the H19/Igf2 cluster [75]. Given the ability of cohesins to tether DNA strands (*i.e.*, sister chromatids after cell’s S-phase) it is possible that cohesins contribute mechanistically to these chromatin contacts on the maternal H19/Igf2 locus. On the paternal allele, where CTCF cannot bind, long-range chromatin interactions are not observed within the cluster, suggesting a state that allows interaction between the 90 kb distant enhancers and *Igf2* (Figure 1D) [73]. Similar interactions involving CTCF have been noted at other loci (Figure 1D).

## 5. Relationship between DNA Methylation and Histone Modifications

Similar to DNA methylation, correlation between multiple histone modifications in various genomic elements, including promoters, have been associated with gene activity and repression, and early studies illustrating this indeed investigated the relationship in the context of imprinted loci [76,77,78,79,80,81,82]. A functional relationship may therefore potentially exist between DNA methylation and histone modifications whereby the acquisition of one may be dependent on, or mutually exclusive, with the other. Indeed, as noted above, MBPs can recruit histone modification enzymes. Well-defined examples of histone modifications that regulate *de novo* DNA methylation are however scarce [83,84]. One very compelling example again comes from the study of genomic imprinting, as discussed below.

DNMT3L lacks a DNA methyltransferase activity, but it is necessary for methylation of DNA in certain situations [85,86] because it forms a complex with DNMT3A and DNMT3B, impacts their activity and contributes to their structural interaction with chromatin [87,88,89,90]. The ability is likely a result of a recently discovered affinity of DNMT3L to histone H3 [90] and this interaction is dependent on the methylation state of H3 at lysine K4—the binding only occurs when the histone is unmethylated hence H3 methylation might shield from DNA methylation [90]. A functional role for H3K4 methylation in modulating DNA methylation came from an imprinting study where Ciccone *et al*. showed that this interaction has important regulatory implications. The group generated mice deficient for a H3K4 demethylase enzyme, KDM1B, which resulted in increased H3K4 methylation in oocytes, where KDM1B is almost exclusively expressed. Consistent with inhibition of the DNMT3L-DNMT3A complex binding to methylated histone H3, DMRs at four imprinted regions that normally acquire DNA methylation in the female germ line were unmethylated in the *Kdm1b* null oocytes and imprinted expression of the corresponding genes was lost in embryos from *Kdm1b* null females (Figure 1E) [91]. These results strongly suggest a functional link between loss of H3K4 methylation and acquisition of DNA methylation, at least at imprinted regions (Figure 1E).

Cedar and Bergman take this further proposing a model of how the bimodal methylation pattern of mammalian genomes may be dependent on this same relationship. They suggest that *de novo* DNA methylation at the blastocyst stage is prevented at particular loci by deposition of H3K4 methylation. They further suggest that H3K4 methyltransferases may be targeted to CpG islands by RNA polymerase II and as a consequence, the DNA methyltransferase machinery containing DNMT3L, cannot access CpG sites in regulatory regions that are CpG islands [84].

H3K9 di- and trimethylation is associated with repressive DNA. DNA methylation is often found at such regions. Furthermore, DNA is globally hypomethylated in mouse ES cells carrying deletion of a H3K9 methyltransferase, G9a [92]. In this case the loss of DNA methylation is not a result of the aberrantly low levels of histone methylation, but rather due to loss of the histone methyltransferase enzyme itself; the DNA methyltransferase machinery interacts with G9a, and this interaction is mediated through a protein domain that is independent of the histone methyltransferase catalytic activity by a SET protein domain. Therefore, in *G9a*^−/−^ mouse ES cells carrying *G9a* transgenes that lack histone methyltransferase activity, e.g., due to a point mutation in the SET domain, DNA methylation levels are partially rescued [93,94]. Regulation of DNA methylation through interaction of the DNMTs with histone modifiers, rather than with the histone modifications themselves, seems to be common and is observed for multiple mammalian histone methyltransferases [95,96,97], as well as in plant [98] and fungal systems [99]. Interestingly, in *G9a*^−/−^ ES cells DNA methylation is lost at some imprinted loci [94,100], but where tested this is not observed in embryos [100,101]. This behaviour at imprints may suggest that ES cell culture is not a faithful model for assessing a requirement for histone modifying enzymes in DNA methylation, but equally might also reflect different properties of imprint-specific maintenance in ES cells compared to *in vivo*.

(**A**) left: In the male germline (sperm), CpG dense regions are generally unmethylated and less dense regions are methylated. In the female germline (oocytes) CpG rich regions are more frequently methylated. This results in multiple differentially methylated regions between the male and female germlines. After fertilization only a small subset of these regions retain differential methylation. Retention of differential methylation at imprinting control regions (diamonds) post-fertilization may therefore be a targeted protection from either demethylation or *de novo* methylation; right: Model; Changes in DNA methylation may be mediated through loss and gain of such protection—when protection is lost (e.g., upper—as a result of factor (black triangle) binding or a histone modification that is non-permissive (red circle) for DNMT binding) CpGs become methylated by the methyltransferase machinery. If protection is gained (lower) the machinery cannot access the CpG sites to maintain methylation and after cell divisions methylation is therefore lost; (**B**) left: The secondary DMR located in the promoter region of the imprinted gene *Gtl2* becomes methylated on the paternal allele after expression is silenced; right: Model; DNA methylation at CpG sites in promoter regions of non-imprinted genes may therefore, at least in some cases, occur after gene silencing; (**C**) left: In the H19/Igf2 imprinted locus, CTCF (red pentagon) binds the H19-ICR, on the unmethylated maternal allele, not the methylated paternal allele; right: Model; Methylation of CpG sites can inhibit protein binding (purple) to DNA; (**D**) left: In the H19-Igf2 imprinted locus CTCF (red pentagon) binds to regions flanking *Igf2* and dimerizes, looping the gene and physically inhibiting its interaction with distal enhancers. On the paternally inherited allele, CTCF does not bind and enhancers are in contact with *Igf2* and the gene is expressed; right: Model; Looping of DNA sequences through the action of CTCF (red pentagon) can separate regions or bring them into contact; (**E**) left: In *Kdm1b^−/−^* (histone methyltransferase) mouse oocytes, imprints are not established at multiple ICRs due to the inhibitory effect of H3K4 methylation on DNMT3L. Histone modification states in WT and *Kdm1b*^−/−^ mice are depicted as green and red circles to signify permissive and non-permissiveness to *de novo* DNA methylation, respectively. In embryos from *Kdm1b*^−/−^ mothers, imprinted expression is lost, and genes are biallelically expressed (*Mest,* depicted) or repressed; right: Model; Histone modifications (red and green circles) can regulate DNA methylation; (**F**) left: In the Igf2r imprinted locus *Igf2r* expression is inhibited by transcriptional interference from the *Airn* lncRNA transcript on the paternal allele. The lncRNA recruits histone modifiers such as G9a (blue) to proximal imprinted genes that contribute to silencing of the imprinted *Slc22a3* in a lineage specific manner, e.g., through deposition of histone marks that are non-permissive for transcription (red circle); upper right: Model; lncRNAs may exert their effects in *trans* at proximal genes. As illustrated, a lncRNA is expressed and silences proximal genes, but not the more distal genes; lower right: Model; In the example provided a lncRNA and a coding gene are expressed from within the same ORF. Transcription of the lncRNA inhibits expression of the coding gene.

## 6. lncRNAs

### 6.1. lncRNAs, Definition, Characterization and Potential Functions

In recent years the roles of long noncoding RNAs (lncRNAs) in regulating genome function have received considerable attention, and are now emerging as a large group of genes with potential functions of fundamental importance for cell biology (for review see [2,102,103,104]). lncRNAs are defined as noncoding RNA transcripts of >200 bp [104]. Transcription of lncRNAs resembles that of mRNA genes; they are transcribed by the same transcriptional machinery and by RNA polymerase II, the transcripts are 5' capped and can be spliced and shuttled to the cytoplasm [102]. The lack of an open reading frame and their size are therefore the only criteria that currently define lncRNAs as a group [102,104]. On basis of high-throughput RNA sequencing experiments, the numbers of lncRNA transcripts have been suggested to range between 5000–15,000 [105,106]. With higher sensitivity, targeted capture experiments have identified lncRNAs that are undetectable by high-throughput technology, suggesting that this range is an underestimate [107]. However, as a result of their loosely defined criteria, lncRNAs as a group may be very heterogeneous. Therefore, the functional roles discussed below may only apply to a subset of their estimated numbers.

Despite the current excitement surrounding “new” roles for lncRNAs, they were shown to regulate genomic imprinting over a decade ago. Multiple potential functions of lncRNAs have been proposed whereby lncRNAs either exert their effects by acting in *trans* or by the act of their transcription in *cis* (transcriptional interference). Both effects have been shown to act at imprinted loci. Some *trans* acting lncRNAs, such as HOTAIR, have been suggested to exert their effects throughout the genome [108], while others, including most imprinted lncRNAs defined to date, act over a limited area surrounding or close to their transcriptional origin. Some lncRNAs may utilize both *cis* and *trans* acting mechanisms. An example is the imprinted Airn lncRNA whose transcription on the paternal chromosome represses *Igf2r* expression in *cis* by transcriptional interference [109], while the Airn RNA molecule itself is also necessary for regulating other genes in the cluster in a *trans*-targeted manner (see below, and Figure 1F [110]). Transcriptional interference is proposed to occur as a result of a collision between the transcriptional machineries of two adjacent or overlapping transcripts which might result in termination of one or both transcriptional events. Alternatively it may occur by promoter occlusion via inhibition of formation of a transcriptional initiation complex due to existing transcription of one transcript through the promoter of another [111].

Functions of *trans* acting lncRNAs have been proposed to fall into the following categories [112]: (1) Decoys: lncRNAs that bind to DNA binding proteins and prevent their interaction with DNA; (2) Scaffolds: lncRNA that function to join two or more proteins into an lncRNA-RNP (ribonucleoprotein) complex; (3) Guides: lncRNAs that bind proteins to guide them to certain genomic locations, e.g., by lending them specificity and/or binding capacity to certain DNA sequences or chromatin states.

### 6.2. lncRNAs in the Epigenetic Control of Genome Function—Lessons from Imprinting

Every cluster of imprinted genes contains at least one lncRNA and these lncRNAs are regulated by DNA methylation. This was demonstrated in experiments where the genes encoding DNA methyltransferases were deleted in mice to gauge effects on imprinting regulation. Promoters for the *Airn*, *Nespas/Gnasxl*, * Snrpn* and *Kcnq1ot1* lncRNA genes lie within the ICR for their respective region and are differentially methylated on the two parental chromosomes. Upon loss of DNMT1, the maintenance methyltransferase, methylation is lost at these ICRs in E10.5 embryos (the genetic manipulation is lethal at later embryonic stages) and *Airn, Nespas/Gnasxl*, * Snrpn* and *Kcnq1ot1* are biallelically expressed, with effects on neighboring imprinted protein coding genes, some of which may lose imprinting as a result of the lncRNA dysregulation [14,113,114]. *Kcnq1ot1* and *Airn* promoters are located in the ICRs, exhibit differential methylation, and, importantly, are located within genes running antisense to them, hence these provide examples of critical regulatory DNA methylation at genomic regions considered by some to have little or no consequence, *i.e.*, intragenic. The existence of other epigenetically regulated elements within genes and acting in this way to potentially regulate lncRNAs, may have very widespread effects on genome function.

Furthermore, imprinted lncRNAs have been demonstrated to be necessary for epigenetic control of genome function, to guide chromatin modifying enzymes in *trans* to specific sites in the genome. This is thought to mediate changes in histone modifications and be associated with changes in transcriptional activity. Although challenging to address experimentally, this function for lncRNAs is currently the topic of much attention. It was studies on imprinted gene regulation at the Igf2r/Airn and Kcnq1/Kcnq1ot1 imprinted clusters that provided examples of this type of regulation [104]. *Kcnq1ot1* and *Airn* are estimated as greater than 100 kb lncRNA transcripts, transcribed in an antisense orientation from within protein coding genes; *Kcnq1ot1* from *Kcnq1* in a 1 Mb imprinted cluster that contains eight maternally expressed protein coding genes, and *Airn* from *Igf2r* in a 400 kb long imprinted cluster that contains three maternally expressed protein coding genes. Both transcripts generate unspliced lncRNAs that are localized in the nucleus [103,115,116]. The ICRs of both genes are methylated on the maternal, but not paternally inherited chromosome, and determine monoallelic expression of the lncRNAs from the paternal allele. In mouse genetic mutants, where the promoters of *Airn* and *Kcnq1ot1* are deleted or their transcripts truncated by insertion of premature polyA sequence into the endogenous genes, biallelic expression of the imprinted protein coding genes occurs within their respective clusters [110,117,118,119]. These results suggested that the lncRNAs or the act of their transcription is necessary for silencing of genes in *cis* (Figure 1F). In addition, several lines of evidence further indicate that lncRNAs guide chromatin modifying enzymes in *trans* to establish repressive histone marks and gene silencing on the paternal allele (Figure 1F): In the Kcnq1 imprinted cluster *Osbpl5*, *Cd81*, *Ascl2* and *Tscc4* are imprinted exclusively in the placenta [40] and so are *Slc22a2* and *Slc22a3* in the Igf2r/Airn cluster [120]. The paternal chromosomes are bound by the histone methyltransferases G9a and/or Ezh-Eed2 in the extraembryonic lineage [40,110], and both Airn and Kcnq1ot1 lncRNAs associate with G9a histone methyltransferase in a lineage specific manner—in placenta but not embryo [110,115]. These results showed that lncRNAs may be a contributing factor for targeting epigenetic marks (Figure 1F) with genetic models being used alongside biochemical approaches to generate a more tractable and comparable experimental paradigm for added robustness. These studies have paved the way for explorations of the roles of multiple other lncRNAs which are found in association with different chromatin modifying enzymes [121,122]. Most recently, the imprinted lncRNA Gtl2/Meg3 has been shown to function in *trans* to target polycomb regulatory complexes in mouse and human stem cells in culture [123].

## 7. Conclusions

The robust genetic approaches applied to the regulation of imprinting have allowed it to be an excellent hypothesis-driven model to investigate and understand the epigenetic control of genome regulation. One of its greatest strengths as a model is that it allows the comparison of differentially expressed alleles of the two inherited copies of a gene with identical sequence within the same cell. Because these two parentally inherited alleles have well-defined different epigenetic states the contributions of these to gene expression can be determined. Since imprinted clusters employ multiple different epigenetic mechanisms, acting through various different mediators (long non-coding RNA, CTCF, *etc*.), this has enabled investigators to explore their hierarchical interactions and relationships with one another. As evidenced by the examples presented here, imprinting has provided insight into some of the most fundamental aspects of a range of epigenetic phenomena and their mediators.

Nevertheless, many important aspects of imprinting and epigenetic control remain to be elucidated. These include: what allows epigenetic marks to be *de novo* targeted differently in the male and female germlines; whether they are modulated by extrinsic or intrinsic signals, for example in the context of development and disease; and how DNA methylation is actively removed during reprogramming and perhaps at other times in development. The mechanisms regulating some of these processes are beginning to emerge where the context of imprinting has contributed; the DNA binding proteins ZFP57 and PGC/Stella have been shown to target and maintain DNA methylation at imprinted clusters during postfertilisation epigenetic reprogramming [19,124] and selective loss of imprinting is necessary for stem cell regulation in the neurogenic niche of the developing mouse [125]. Whether we can apply more generally what we learn from these mechanisms—for example about the general targeting of epigenetic states or the dynamic changes in epigenetic state in specific cellular niches—remains to be determined. It is likely that future studies, addressing these and other similarly fundamental questions in the context of imprinting will continue to add new layers to our understanding of genome regulation and the epigenetic control of genome function more widely.

## References

[1] Miranda T.B., Jones P.A. (2007). DNA methylation: The nuts and bolts of repression. J. Cell. Physiol..

[2] Gibney E.R., Nolan C.M. (2010). Epigenetics and gene expression. Heredity (Edinb)..

[3] Goll M.G., Bestor T.H. (2005). Eukaryotic cytosine methyltransferases. Annu. Rev. Biochem..

[4] Smallwood S.A., Kelsey G. (2012). *De novo* DNA methylation: A germ cell perspective. Trends Genet..

[5] Kaneda M., Okano M., Hata K., Sado T., Tsujimoto N., Li E., Sasaki H. (2004). Essential role for *de novo* DNA methyltransferase Dnmt3a in paternal and maternal imprinting. Nature.

[6] Gu T.-P., Guo F., Yang H., Wu H.-P., Xu G.-F., Liu W., Xie Z.-G., Shi L., He X., Jin S. (2011). The role of Tet3 DNA dioxygenase in epigenetic reprogramming by oocytes. Nature.

[7] Berger S.L. (2007). The complex language of chromatin regulation during transcription. Nature.

[8] Ferguson-Smith A.C. (2011). Genomic imprinting: The emergence of an epigenetic paradigm. Nat. Rev. Genet..

[9] Edwards C.A., Ferguson-Smith A.C. (2007). Mechanisms regulating imprinted genes in clusters. Curr. Opin. Cell Biol..

[10] McGrath J., Solter D. (1984). Completion of mouse embryogenesis requires both the maternal and paternal genomes. Cell.

[11] Surani M.A.H., Barton S.C., Norris M.L. (1984). Development of reconstituted mouse eggs suggests imprinting of the genome during gametogenesis. Nature.

[12] Ferguson-Smith A.C., Sasaki H., Cattanach B.M., Surani M.A. (1993). Parental-origin-specific epigenetic modification of the mouse *H19* gene. Nature.

[13] Stöger R., Kubicka P., Liu C.-G., Kafri T., Razin A., Cedar H., Barlow D.P. (1993). Maternal-specific methylation of the imprinted mouse *Igf2r* locus identifies the expressed locus as carrying the imprinting signal. Cell.

[14] Li E., Beard C., Jaenisch R. (1993). Role for DNA methylation in genomic imprinting. Nature.

[15] Smallwood S.A., Tomizawa S., Krueger F., Ruf N., Carli N., Segonds-Pichon A., Sato S., Hata K., Andrews S.R., Kelsey G. (2011). Dynamic CpG island methylation landscape in oocytes and preimplantation embryos. Nat. Genet..

[16] Kelsey G., Feil R. (2013). New insights into establishment and maintenance of DNA methylation imprints in mammals. Phil. Trans. R. Soc. B.

[17] Kobayashi H., Sakurai T., Imai M., Takahashi N., Fukuda A., Yayoi O., Sato S., Nakabayashi K., Hata K., Sotomaru Y. (2012). Contribution of intragenic DNA methylation in mouse gametic DNA methylomes to establish oocyte-specific heritable marks. PLoS Genet..

[18] Smith Z.D., Chan M.M., Mikkelsen T.S., Gu H., Gnirke A., Regev A., Meissner A. (2012). A unique regulatory phase of DNA methylation in the early mammalian embryo. Nature.

[19] Li X., Ito M., Zhou F., Youngson N., Zuo X., Leder P., Ferguson-Smith A.C. (2008). A maternal-zygotic effect gene, *Zfp57*, maintains both maternal and paternal imprints. Dev. Cell.

[20] Groudine M., Eisenman R., Weintraub H. (1981). Chromatin structure of endogenous retroviral genes and activation by an inhibitor of DNA methylation. Nature.

[21] Sutter D., Doerfler W. (1980). Methylation of integrated adenovirus type 12 DNA sequences in transformed cells is inversely correlated with viral gene expression. Proc. Natl. Acad. Sci. USA.

[22] Desrosiers R.C., Mulder C., Fleckenstein B. (1979). Methylation of *Herpesvirus saimiri* DNA in lymphoid tumor cell lines. Proc. Natl. Acad. Sci. USA.

[23] Cohen J.C. (1980). Methylation of milk-borne and genetically transmitted mouse mammary tumor virus proviral DNA. Cell.

[24] Guntaka R.V., Rao P.Y., Mitsialis S.A., Katz R. (1980). Modification of avian sarcoma proviral DNA sequences in nonpermissive XC cells but not in permissive chicken cells. J. Virol..

[25] Van der Ploeg L.H.T., Flavell R.A. (1980). DNA methylation in the human globin locus in erythroid and nonerythroid tissues. Cell.

[26] McGhee J.D., Ginder G.D. (1979). Specific DNA methylation sites in the vicinity of the chicken beta-globin genes. Nature.

[27] Kuo M.T., Mandel J.L., Chambon P. (1979). DNA methylation: Correlation with DNase I sensitivity of chicken ovalbumun and conalbumin chromatin. Nucleic Acids Res..

[28] Mandel J.L., Chambon P. (1979). DNA methylation: Organ specific variations in the methylation pattern within and around ovalbumin and other chicken genes. Nucleic Acids Res..

[29] Bird A.P., Taggart M.H., Smith B.A. (1979). Methylated and unmethylated DNA compartments in the sea urchin genome. Cell.

[30] Bird A., Taggart M., Macleod D. (1981). Loss of rDNA methylation accompanies the onset of ribosomal gene activity in early development of *X. laevis*. Cell.

[31] Vardimon L., Kressmann A., Cedar H., Maechler M., Doerfler W. (1982). Expression of a cloned adenovirus gene is inhibited by *in vitro* methylation. Proc. Natl. Acad. Sci. USA.

[32] Stein R., Razin A., Cedar H. (1982). *In vitro* methylation of the hamster adenine phosphoribosyltransferase gene inhibits its expression in mouse L cells. Proc. Natl. Acad. Sci. USA.

[33] Ball M.P., Li J.B., Gao Y., Lee J.-H., LeProust E.M., Park I.-H., Xie B., Daley G.Q., Church G.M. (2009). Targeted and genome-scale strategies reveal gene-body methylation signatures in human cells. Nat. Biotechnol..

[34] Deng J., Shoemaker R., Xie B., Gore A., LeProust E.M., Antosiewicz-Bourget J., Egli D., Maherali N., Park I.-H., Yu J. (2009). Targeted bisulfite sequencing reveals changes in DNA methylation associated with nuclear reprogramming. Nat. Biotechnol..

[35] Rauch T.A., Wu X., Zhong X., Riggs A.D., Pfeifer G.P. (2009). A human B cell methylome at 100-base pair resolution. Proc. Natl. Acad. Sci. USA.

[36] Trowbridge J.J., Orkin S.H. (2010). DNA methylation in adult stem cells. Epigenetics.

[37] Razin A., Cedar H. (1991). DNA methylation and gene expression. Microbiol. Rev..

[38] Sato S., Yoshida W., Soejima H., Nakabayashi K., Hata K. (2011). Methylation dynamics of IG-DMR and *Gtl2*-DMR during murine embryonic and placental development. Genomics.

[39] Bhogal B., Arnaudo A., Dymkowski A., Best A., Davis T.L. (2004). Methylation at mouse *Cdkn1c* is acquired during postimplantation development and functions to maintain imprinted expression. Genomics.

[40] Umlauf D., Goto Y., Cao R., Cerqueira F., Wagschal A., Zhang Y., Feil R. (2004). Imprinting along the *Kcnq1* domain on mouse chromosome 7 involves repressive histone methylation and recruitment of Polycomb group complexes. Nat. Genet..

[41] Sasaki H., Ferguson-Smith A.C., Shum A.S.W., Barton S.C., Surani M.A. (1995). Temporal and spatial regulation of H19 imprinting in normal and uniparental mouse embryos. Development.

[42] Lerchner W., Barlow D.P. (1997). Paternal repression of the imprinted mouse *Igf2r* locus occurs during implantation and is stable in all tissues of the post-implantation mouse embryo. Mech. Dev..

[43] Stadler M.B., Murr R., Burger L., Ivanek R., Lienert F., Schöler A., van Nimwegen E., Wirbelauer C., Oakeley E.J., Gaidatzis D. (2011). DNA-binding factors shape the mouse methylome at distal regulatory regions. Nature.

[44] Klose R.J., Bird A.P. (2006). Genomic DNA methylation: The mark and its mediators. Trends Biochem. Sci..

[45] Meehan R.R., Lewis J.D., McKay S., Kleiner E.L., Bird A.P. (1989). Identification of a mammalian protein that binds specifically to DNA containing methylated CpGs. Cell.

[46] Lewis J.D., Meehan R.R., Henzel W.J., Maurer-Fogy I., Jeppesen P., Klein F., Bird A. (1992). Purification, sequence, and cellular localization of a novel chromosomal protein that binds to methylated DNA. Cell.

[47] Hendrich B., Bird A. (1998). Identification and characterization of a family of mammalian methyl-CpG binding proteins. Mol. Cell. Biol..

[48] Hendrich B., Tweedie S. (2003). The methyl-CpG binding domain and the evolving role of DNA methylation in animals. Trends Genet..

[49] Bird A.P., Wolffe A.P. (1999). Methylation-induced repression—Belts, braces, and chromatin. Cell.

[50] Jones P.L., Veenstra G.J.C., Wade P.A., Vermaak D., Kass S.U., Landsberger N., Strouboulis J., Wolffe A.P. (1998). Methylated DNA and MeCP2 recruit histone deacetylase to repress transcription. Nat. Genet..

[51] Nan X., Ng H.-H., Johnson C.A., Laherty C.D., Turner B.M., Eisenman R.N., Bird A. (1998). Transcriptional repression by the methyl-CpG-binding protein MeCP2 involves a histone deacetylase complex. Nature.

[52] Ng H.-H., Zhang Y., Hendrich B., Johnson C.A., Turner B.M., Erdjument-Bromage H., Tempst P., Reinberg D., Bird A. (1999). MBD2 is a transcriptional repressor belonging to the MeCP1 histone deacetylase complex. Nat. Genet..

[53] Wade P.A., Gegonne A., Jones P.L., Ballestar E., Aubry F., Wolffe A.P. (1999). Mi-2 complex couples DNA methylation to chromatin remodelling and histone deacetylation. Nat. Genet..

[54] Zhang Y., Ng H.-H., Erdjument-Bromage H., Tempst P., Bird A., Reinberg D. (1999). Analysis of the NuRD subunits reveals a histone deacetylase core complex and a connection with DNA methylation. Genes Dev..

[55] Sarraf S.A., Stancheva I. (2004). Methyl-CpG binding protein MBD1 couples histone H3 methylation at lysine 9 by SETDB1 to DNA replication and chromatin assembly. Mol. Cell.

[56] Prendergast G.C., Lawe D., Ziff E.B. (1991). Association of Myn, the murine homolog of Max, with c-Myc stimulates methylation-sensitive DNA binding and Ras cotransformation. Cell.

[57] Watt F., Molloy P.L. (1988). Cytosine methylation prevents binding to DNA of a HeLa cell transcription factor required for optimal expression of the adenovirus major late promoter. Genes Dev..

[58] Comb M., Goodman H.M. (1990). CpG methylation inhibits proenkephalin gene expression and binding of the transcription factor AP-2. Nucleic Acids Res..

[59] DeChiara T.M., Robertson E.J., Efstratiadis A. (1991). Parental imprinting of the mouse insulin-like growth factor II gene. Cell.

[60] Thorvaldsen J.L., Duran K.L., Bartolomei M.S. (1998). Deletion of the *H19* differentially methylated domain results in loss of imprinted expression of *H19* and *Igf2*. Genes Dev..

[61] Tremblay K.D., Duran K.L., Bartolomei M.S. (1997). A 5' 2-kilobase-pair region of the imprinted mouse *H19* gene exhibits exclusive paternal methylation throughout development. Mol. Cell. Biol..

[62] Szabó P.E., Tang S.-H.E., Rentsendorj A., Pfeifer G.P., Mann J.R. (2000). Maternal-specific footprints at putative CTCF sites in the *H19* imprinting control region give evidence for insulator function. Curr. Biol..

[63] Kanduri C., Pant V., Loukinov D., Pugacheva E., Qi C.-F., Wolffe A., Ohlsson R., Lobanenkov V.V. (2000). Functional association of CTCF with the insulator upstream of the *H19* gene is parent of origin-specific and methylation-sensitive. Curr. Biol..

[64] Hark A.T., Schoenherr C.J., Katz D.J., Ingram R.S., Levorse J.M., Tilghman S.M. (2000). CTCF mediates methylation-sensitive enhancer-blocking activity at the *H19/Igf2* locus. Nature.

[65] Bell A.C., Felsenfeld G. (2000). Methylation of a CTCF-dependent boundary controls imprinted expression of the *Igf2* gene. Nature.

[66] Webber A.L., Ingram R.S., Levorse J.M., Tilghman S.M. (1998). Location of enhancers is essential for the imprinting of *H19* and *Igf2* genes. Nature.

[67] Leighton P.A., Saam J.R., Ingram R.S., Stewart C.L., Tilghman S.M. (1995). An enhancer deletion affects both *H19* and *Igf2* expression. Genes Dev..

[68] Phillips J.E., Corces V.G. (2009). CTCF: Master weaver of the genome. Cell.

[69] Pant V., Kurukuti S., Pugacheva E., Shamsuddin S., Mariano P., Renkawitz R., Klenova E., Lobanenkov V., Ohlsson R. (2004). Mutation of a single CTCF target site within the *H19* imprinting control region leads to loss of *Igf2* imprinting and complex patterns of *de novo* methylation upon maternal inheritance. Mol. Cell. Biol..

[70] Yusufzai T.M., Tagami H., Nakatani Y., Felsenfeld G. (2004). CTCF tethers an insulator to subnuclear sites, suggesting shared insulator mechanisms across species. Mol. Cell.

[71] Yoon Y.S., Jeong S., Rong Q., Park K.-Y., Chung J.H., Pfeifer K. (2007). Analysis of the *H19ICR* insulator. Mol. Cell. Biol..

[72] Li T., Hu J.-F., Qiu X., Ling J., Chen H., Wang S., Hou A., Vu T.H., Hoffman A.R. (2008). CTCF regulates allelic expression of *Igf2* by orchestrating a promoter-polycomb repressive complex 2 intrachromosomal loop. Mol. Cell. Biol..

[73] Kurukuti S., Tiwari V.K., Tavoosidana G., Pugacheva E., Murrell A., Zhao Z., Lobanenkov V., Reik W., Ohlsson R. (2006). CTCF binding at the *H19* imprinting control region mediates maternally inherited higher-order chromatin conformation to restrict enhancer access to *Igf2*. Proc. Natl. Acad. Sci. USA.

[74] Murrell A., Heeson S., Reik W. (2004). Interaction between differentially methylated regions partitions the imprinted genes *Igf2* and *H19* into parent-specific chromatin loops. Nat. Genet..

[75] Wendt K.S., Yoshida K., Itoh T., Bando M., Koch B., Schirghuber E., Tsutsumi S., Nagae G., Ishihara K., Mishiro T. (2008). Cohesin mediates transcriptional insulation by CCCTC-binding factor. Nature.

[76] Hon G.C., Hawkins R.D., Ren B. (2009). Predictive chromatin signatures in the mammalian genome. Hum. Mol. Genet..

[77] Ernst J., Kheradpour P., Mikkelsen T.S., Shoresh N., Ward L.D., Epstein C.B., Zhang X., Wang L., Issner R., Coyne M. (2011). Mapping and analysis of chromatin state dynamics in nine human cell types. Nature.

[78] Li B., Carey M., Workman J.L. (2007). The role of chromatin during transcription. Cell.

[79] Hon G., Wang W., Ren B. (2009). Discovery and annotation of functional chromatin signatures in the human genome. PLoS Comput. Biol..

[80] Zhou V.W., Goren A., Bernstein B.E. (2011). Charting histone modifications and the functional organization of mammalian genomes. Nat. Rev. Genet..

[81] Grandjean V., O’Neill L., Sado T., Turner B., Ferguson-Smith A. (2001). Relationship between DNA methylation, histone H4 acetylation and gene expression in the mouse imprinted *Igf2-H19* domain. FEBS Lett..

[82] Pedone P.V., Pikaart M.J., Cerrato F., Vernucci M., Ungaro P., Bruni C.B., Riccio A. (1999). Role of histone acetylation and DNA methylation in the maintenance of the imprinted expression of the *H19* and *Igf2* genes. FEBS Lett..

[83] Chen T. (2011). Mechanistic and functional links between histone methylation and DNA methylation. Prog. Mol. Biol. Transl. Sci..

[84] Cedar H., Bergman Y. (2009). Linking DNA methylation and histone modification: Patterns and paradigms. Nat. Rev. Genet..

[85] Bourc’his D., Xu G.-L., Lin C.-S., Bollman B., Bestor T.H. (2001). Dnmt3L and the establishment of maternal genomic imprints. Science.

[86] Bourc’his D., Bestor T.H. (2004). Meiotic catastrophe and retrotransposon reactivation in male germ cells lacking Dnmt3L. Nature.

[87] Jia D., Jurkowska R.Z., Zhang X., Jeltsch A., Cheng X. (2007). Structure of Dnmt3a bound to Dnmt3L suggests a model for *de novo* DNA methylation. Nature.

[88] Margot J.B., Ehrenhofer-Murray A.E., Leonhardt H. (2003). Interactions within the mammalian DNA methyltransferase family. BMC Mol. Biol..

[89] Suetake I., Shinozaki F., Miyagawa J., Takeshima H., Tajima S. (2004). DNMT3L stimulates the DNA methylation activity of Dnmt3a and Dnmt3b through a direct interaction. J. Biol. Chem..

[90] Ooi S.K.T., Qiu C., Bernstein E., Li K., Jia D., Yang Z., Erdjument-Bromage H., Tempst P., Lin S.-P., Allis C.D. (2007). DNMT3L connects unmethylated lysine 4 of histone H3 to *de novo* methylation of DNA. Nature.

[91] Ciccone D.N., Su H., Hevi S., Gay F., Lei H., Bajko J., Xu G., Li E., Chen T. (2009). KDM1B is a histone H3K4 demethylase required to establish maternal genomic imprints. Nature.

[92] Ikegami K., Iwatani M., Suzuki M., Tachibana M., Shinkai Y., Tanaka S., Greally J.M., Yagi S., Hattori N., Shiota K. (2007). Genome-wide and locus-specific DNA hypomethylation in G9a deficient mouse embryonic stem cells. Genes Cells.

[93] Tachibana M., Matsumura Y., Fukuda M., Kimura H., Shinkai Y. (2008). G9a/GLP complexes independently mediate H3K9 and DNA methylation to silence transcription. EMBO J..

[94] Dong K.B., Maksakova I.A., Mohn F., Leung D., Appanah R., Lee S., Yang H.W., Lam L.L., Mager D.L., Schübeler D. (2008). DNA methylation in ES cells requires the lysine methyltransferase G9a but not its catalytic activity. EMBO J..

[95] Lehnertz B., Ueda Y., Derijck A.A.H.A., Braunschweig U., Perez-Burgos L., Kubicek S., Chen T., Li E., Jenuwein T., Peters A.H.F.M. (2003). Suv39h-mediated histone H3 lysine 9 methylation directs DNA methylation to major satellite repeats at pericentric heterochromatin. Curr. Biol..

[96] Viré E., Brenner C., Deplus R., Blanchon L., Fraga M., Didelot C., Morey L., van Eynde A., Bernard D., Vanderwinden J.-M. (2006). The Polycomb group protein EZH2 directly controls DNA methylation. Nature.

[97] Li H., Rauch T., Chen Z.-X., Szabó P.E., Riggs A.D., Pfeifer G.P. (2006). The histone methyltransferase SETDB1 and the DNA methyltransferase DNMT3A interact directly and localize to promoters silenced in cancer cells. J. Biol. Chem..

[98] Jackson J.P., Lindroth A.M., Cao X., Jacobsen S.E. (2002). Control of CpNpG DNA methylation by the KRYPTONITE histone H3 methyltransferase. Nature.

[99] Freitag M., Hickey P.C., Khlafallah T.K., Read N.D., Selker E.U. (2004). HP1 is essential for DNA methylation in *Neurospora*. Mol. Cell.

[100] Xin Z., Tachibana M., Guggiari M., Heard E., Shinkai Y., Wagstaff J. (2003). Role of histone methyltransferase G9a in CpG methylation of the Prader-Willi syndrome imprinting center. J. Biol. Chem..

[101] Wagschal A., Sutherland H.G., Woodfine K., Henckel A., Chebli K., Schulz R., Oakey R.J., Bickmore W.A., Feil R. (2008). G9a histone methyltransferase contributes to imprinting in the mouse placenta. Mol. Cell. Biol..

[102] Mercer T.R., Mattick J.S. (2013). Structure and function of long noncoding RNAs in epigenetic regulation. Nat. Struct. Mol. Biol..

[103] Mohammad F., Mondal T., Kanduri C. (2009). Epigenetics of imprinted long noncoding RNAs. Epigenetics.

[104] Ponting C.P., Oliver P.L., Reik W. (2009). Evolution and functions of long noncoding RNAs. Cell.

[105] Derrien T., Johnson R., Bussotti G., Tanzer A., Djebali S., Tilgner H., Guernec G., Martin D., Merkel A., Knowles D.G. (2012). The GENCODE v7 catalog of human long noncoding RNAs: Analysis of their gene structure, evolution, and expression. Genome Res..

[106] Cabili M.N., Trapnell C., Goff L., Koziol M., Tazon-Vega B., Regev A., Rinn J.L. (2011). Integrative annotation of human large intergenic noncoding RNAs reveals global properties and specific subclasses. Genes Dev..

[107] Mercer T.R., Gerhardt D.J., Dinger M.E., Crawford J., Trapnell C., Jeddeloh J.A., Mattick J.S., Rinn J.L. (2012). Targeted RNA sequencing reveals the deep complexity of the human transcriptome. Nat. Biotechnol..

[108] Chu C., Qu K., Zhong F.L., Artandi S.E., Chang H.Y. (2011). Genomic maps of long noncoding RNA occupancy reveal principles of RNA-chromatin interactions. Mol. Cell.

[109] Latos P.A., Pauler F.M., Koerner M.V, Senergin H.B., Hudson Q.J., Stocsits R.R., Allhoff W., Stricker S.H., Klement R.M., Warczok K.E. (2012). *Airn* transcriptional overlap, but not its lncRNA products, induces imprinted *Igf2r* silencing. Science.

[110] Nagano T., Mitchell J.A., Sanz L.A., Pauler F.M., Ferguson-Smith A.C., Feil R., Frase P. (2008). The Air noncoding RNA epigenetically silences transcription by targeting G9a to chromatin. Science.

[111] Osato N., Suzuki Y., Ikeo K., Gojobori T. (2007). Transcriptional interferences in *cis* natural antisense transcripts of humans and mice. Genetics.

[112] Rinn J.L., Chang H.Y. (2012). Genome regulation by long noncoding RNAs. Annu. Rev. Biochem..

[113] Howell C.Y., Bestor T.H., Ding F., Latham K.E., Mertineit C., Trasler J.M., Chaillet J.R. (2001). Genomic imprinting disrupted by a maternal effect mutation in the *Dnmt1* gene. Cell.

[114] Lewis A., Mitsuya K., Umlauf D., Smith P., Dean W., Walter J., Higgins M., Feil R., Reik W. (2004). Imprinting on distal chromosome 7 in the placenta involves repressive histone methylation independent of DNA methylation. Nat. Genet..

[115] Pandey R.R., Mondal T., Mohammad F., Enroth S., Redrup L., Komorowski J., Nagano T., Mancini-DiNardo D., Kanduri C. (2008). *Kcnq1ot1* antisense noncoding RNA mediates lineage-specific transcriptional silencing through chromatin-level regulation. Mol. Cell.

[116] Seidl C.I.M., Stricker S.H., Barlow D.P. (2006). The imprinted *Air* ncRNA is an atypical RNAPII transcript that evades splicing and escapes nuclear export. EMBO J..

[117] Shin J.-Y., Fitzpatrick G.V., Higgins M.J. (2008). Two distinct mechanisms of silencing by the KvDMR1 imprinting control region. EMBO J..

[118] Mancini-DiNardo D., Steele S.J.S., Levorse J.M., Ingram R.S., Tilghman S.M. (2006). Elongation of the *Kcnq1ot1* transcript is required for genomic imprinting of neighboring genes. Genes Dev..

[119] Sleutels F., Zwart R., Barlow D.P. (2002). The non-coding *Air* RNA is required for silencing autosomal imprinted genes. Nature.

[120] Zwart R., Sleutels F., Wutz A., Schinkel A.H., Barlow D.P. (2001). Bidirectional action of the *Igf2r* imprint control element on upstream and downstream imprinted genes. Genes Dev..

[121] Khalil A.M., Guttman M., Huarte M., Garber M., Raj A., Morales R.D., Thomas K., Presser A., Bernstein B.E., van Oudenaarden A. (2009). Many human large intergenic noncoding RNAs associate with chromatin-modifying complexes and affect gene expression. Proc. Natl. Acad. Sci. USA.

[122] Guttman M., Donaghey J., Carey B.W., Garber M., Grenier J.K., Munson G., Young G., Lucas A.B., Ach R., Bruhn L. (2011). lincRNAs act in the circuitry controlling pluripotency and differentiation. Nature.

[123] Kaneko S., Bonasio R., Saldaña-Meyer R., Yoshida T., Son J., Nishino K., Umezawa A., Reinberg D. (2014). Interactions between JARID2 and noncoding RNAs regulate PRC2 recruitment to chromatin. Mol. Cell.

[124] Nakamura T., Arai Y., Umehara H., Masuhara M., Kimura T., Taniguchi H., Sekimoto T., Ikawa M., Yoneda Y., Okabe M. (2007). PGC7/Stella protects against DNA demethylation in early embryogenesis. Nat. Cell Biol..

[125] Ferrón S.R., Charalambous M., Radford E., McEwen K., Wildner H., Hind E., Morante-Redolat J.M., Laborda J., Guillemot F., Bauer S.R. (2011). Postnatal loss of *Dlk1* imprinting in stem cells and niche astrocytes regulates neurogenesis. Nature.

